# Hybrid Metal-Dielectric Nano-Aperture Antenna for Surface Enhanced Fluorescence

**DOI:** 10.3390/ma11081435

**Published:** 2018-08-14

**Authors:** Guowei Lu, Jianning Xu, Te Wen, Weidong Zhang, Jingyi Zhao, Aiqin Hu, Grégory Barbillon, Qihuang Gong

**Affiliations:** 1 State Key Laboratory for Mesoscopic Physics & Collaborative Innovation Center of Quantum Matter, School of Physics, Peking University, Beijing 100871, China; jnxu@pku.edu.cn (J.X.); wente@pku.edu.cn (T.W.); weidongzhang@pku.edu.cn (W.Z.); jingyi.zhao@pku.edu.cn (J.Z.); aiqinhu@pku.edu.cn (A.H.); qhgong@pku.edu.cn (Q.G.); 2 Collaborative Innovation Center of Extreme Optics, Shanxi University, Taiyuan 030006, China; 3 EPF—École d’ingénieurs, 3 bis rue Lakanal, 92330 Sceaux, France; gregory.barbillon@epf.fr

**Keywords:** plasmonics, nano-aperture, surface-enhanced fluorescence, antenna, hybrid

## Abstract

A hybrid metal-dielectric nano-aperture antenna is proposed for surface-enhanced fluorescence applications. The nano-apertures that formed in the composite thin film consist of silicon and gold layers. These were numerically investigated in detail. The hybrid nano-aperture shows a more uniform field distribution within the apertures and a higher antenna quantum yield than pure gold nano-apertures. The spectral features of the hybrid nano-apertures are independent of the aperture size. This shows a high enhancement effect in the near-infrared region. The nano-apertures with a dielectric gap were then demonstrated theoretically for larger enhancement effects. The hybrid nano-aperture is fully adaptable to large-scale availability and reproducible fabrication. The hybrid antenna will improve the effectiveness of surface-enhanced fluorescence for applications, including sensitive biosensing and fluorescence analysis.

## 1. Introduction

Metallic nanostructures can be used as optical nanoantennas and they have attracted increasing attention for plasmon-enhanced spectroscopy and sensitive molecular fluorescence detection [1,2,3]. Nanoantennas can operate beyond the light diffraction limit and have been successfully implemented for single molecule analytical approaches, both in vitro and in vivo. One such design—nano-apertures formed in a metallic film also known as zero-mode waveguides—is an intuitive way to demonstrate the advantages of optical antennae. They offer localized enhancement of excitation light, modification of the fluorescence signal, and suppression of emission from species located outside the apertures [4,5,6,7]. Biophotonic applications of nanoantennas require the efficient enhancement of molecule fluorescence [3,7]. Unfortunately, metallic nanostructures, including nano-apertures antenna, usually suffer from serious absorption and scattering losses that would lead to low quantum yields and even fluorescence quenching [8,9,10]. For instance, due to localized surface plasmon (LSP), a typical nano-aperture can enable sub-wavelength confinement of the optical field at the side corner of holes, resulting in a highly localized excitation light field. The emission quantum efficiency is very low due to metal loss. This can ultimately quench the molecule fluorescence signal at the side corner [4,11].

Photonic structures, such optical micro-cavity or dielectric nanoparticles, have a high quantum yield due to low material loss, but the excitation field enhancement does not compare with the metallic nanostructures [12,13,14]. Meanwhile, hybrid plasmonic-photonic structures can dramatically increase the Purcell factor or achieve efficient waveguides over the uncoupled photonic and plasmonic components [15,16,17,18]. These hybrid systems have been reported in various systems and utilize both the highly localized plasmons and the low-loss photonic modes for Fano resonances, strong coupling, Raman scattering, and spontaneous emission [19,20,21,22]. The concept of hybrid photonic-plasmonic structures has not yet been reported for antennae with a nano-aperture geometry. This aperture antenna geometry is a powerful technology for studying single-molecule real-time dynamics of biological systems, and it is necessary to explore nano-apertures with hybrid configurations to optimize molecular fluorescence.

Here, we report on a hybrid metal-dielectric nano-aperture antenna for surface enhanced fluorescence applications. The nano-apertures formed in a composite film consisting of silicon and gold layers on a glass substrate. They have a more uniform field distribution and a higher antenna quantum yield than pure gold nano-apertures. We also investigated the dependence of surface-enhanced fluorescence on the aperture size and the thickness of the dielectric layer. Furthermore, nano-apertures with a dielectric gap are proposed and shown theoretically to offer better enhancement. The hybrid dielectric plasmonic nano-aperture antenna has better performance. In addition, we discuss several ways to further improve the surface enhancement effects, such as combining this scheme with periodic gratings or the use of lossless high-index dielectric materials.

## 2. Materials and Methods

To investigate the surface-enhanced fluorescence performances of the nano-aperture antennas, finite-difference time-domain (FDTD) was used to calculate the electromagnetic features of the nanostructures, including electromagnetic field distribution, Purcell factor, and antenna quantum efficiency [23,24,25]. The FDTD is a mature method that has been extensively employed to study both the near- and far-field electromagnetic responses of the nanostructures with different arbitrary shapes [26,27]. This method permits the computation of: (i) the electromagnetic field distribution of the nanostructure surroundings and (ii) the electromagnetic flux of a dipole source near metallic nanostructures. In calculations, the optical dielectric function of the gold and silicon materials are modeled while using a Drude-Lorentz dispersion function [28,29]. In all of the calculations, the refractive index of the surrounding media is taken to be 1.33 for water and 1.49 for silica glass. The geometry origin point is set at the center of the water-glass interface.

**Figure 1:**
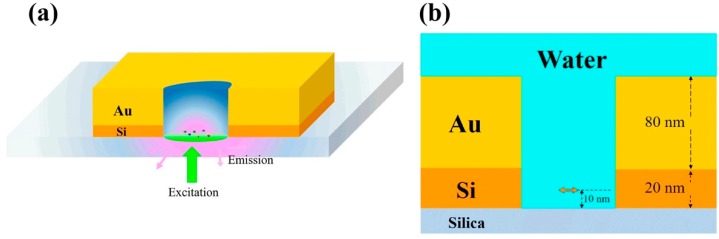
(**a**) Three-dimensional illustration of Au/Si hybrid nano-aperture used as antenna; (**b**) cross-sectional scheme of the hybrid structure consisting of an 80-nm Au layer on the glass separated by a 20-nm Si layer. The diameter of the nano-aperture is 40 nm, and the dipole is 10 nm above the glass surface. The background is water with a refractive index of 1.33.

The scheme of the nano-aperture antenna is shown in Figure 1. It is formed in a thin film composed of Au and Si layers on a silica substrate. A single point dipole orienting along the *z*-direction is placed within the nano-aperture. During calculations, the horizontal dipole is usually positioned at the central point along the *x*-direction and a position 10 nm above the silica glass along the *z*-direction, i.e., position (0, 0, and 10 nm). The emission of a single point dipole source was referred to as a single quantum emitter. The optical antenna quantum yield is the ratio of P_rad_, represents the energy that reaches the far field, to P_tot_ the total power dissipated by the emitter. P_rad_ was obtained by integrating the Poynting vector over closed surfaces that contain the nanoantenna and dipolar source, while P_tot_ was obtained over closed surfaces containing the dipolar source only. We note that the antenna quantum yield is actually the efficiency of radiation of an emitter interacting with an antenna, and the radiation efficiency is dependent on the emitter’s distance and orientation with respect to the nanostructures. During the bottom collection scheme, i.e., P_rad-glass_, a collecting planar monitor under a 10-nm water-glass interface with a region of 2 µm × 2 µm in the *xy*-plane is applied to integrate far field radiation. We compute these quantities by considering the power that is emitted by a classical oscillating dipole and normalize them with respect to the case without any structures [24,30]. Moreover, the FDTD method can calculate the electromagnetic field distribution under plane wave illumination of classical light from the bottom to estimate the field enhancement and thus compare the fluorescence enhancement effects.

## 3. Results and Discussion

**Figure 2:**
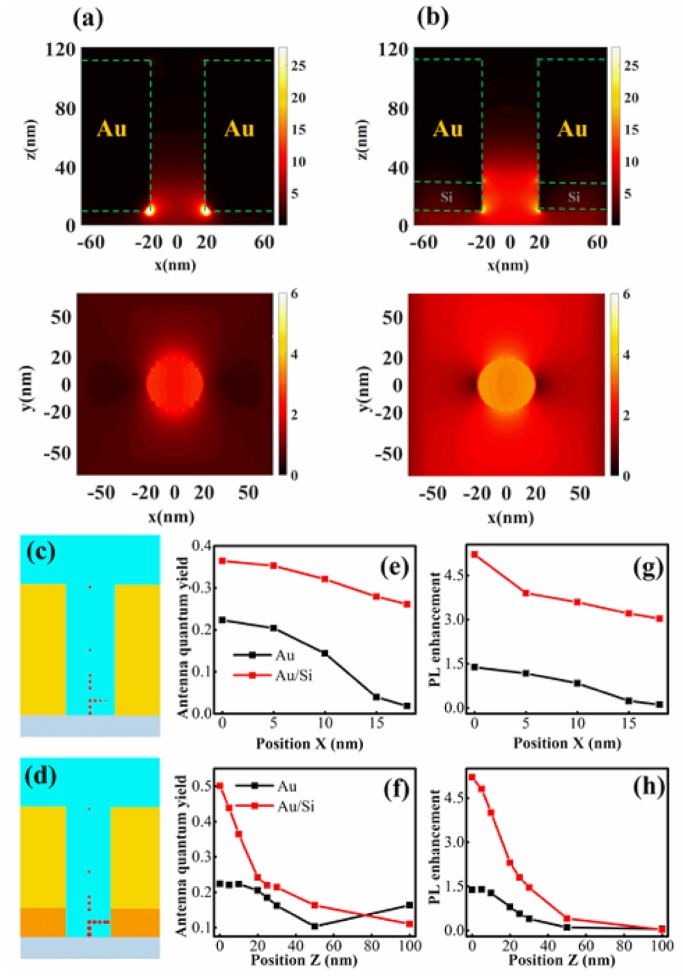
Electric field distribution |E/E_0_|^2^ in the *xz*-plane and *xy*-plane for (**a**) Au nano-aperture antenna at 573 nm and (**b**) Au/Si hybrid nano-aperture antenna at 785 nm illuminated by a plan wave with *x*-polarization from the bottom. The position-dependent quantum yields (the sizes of red disks represent the relative value of quantum yield at the positions correspondingly) (**c**) at 660 nm for the Au nano-aperture antenna; (**d**) at 814 nm for Au/Si hybrid nano-aperture antenna; (**e**,**f**) show quantum yields at different *x*- and *z*-positions indicated in (**c**,**d**), correspondingly. Panels (**g**,**h**) show photoluminescence (PL) enhancements at different *x*- and *z*-positions indicated in (**c**,**d**), correspondingly.

For the nano-aperture structures that are studied here, we first calculate the electromagnetic field distributions and plot the cross-sections of the electromagnetic field distribution in both the *xz*-plane and the *xy*-plane (Figure 2a,b, respectively). In the gold aperture, it is clear that the maximum field enhancement occurs at the corners between the Au layer and the glass. The field intensity is low at the center. Figure 2b shows that the electrical field distribution of the Au/Si hybrid nano-aperture is more uniform than that shown in Figure 2a. Meanwhile, the field of the hybrid aperture presents a larger enhancement than the gold aperture in the center region. The antenna quantum yields of different apertures are also calculated for comparison.

We found that the antenna quantum yield is dependent on the emitter’s position within the nano-aperture structures. Figure 2c,d illustrate the emitter’s position and the relative intensity of the antenna quantum yields for an emitter at 660 nm in the Au nano-aperture or for an emitter at 814 nm in the Au/Si nano-aperture, respectively. Figure 2e shows that we fix the emitter at 10 nm above the glass surface (i.e., 10 nm in the *z*-axis), but at different positions along the *x*-direction. The antenna quantum yield of the gold nano-aperture decreases when the dipole is closer to the gold surface. The quantum yield becomes very low when the emitter is close to the gold surface at the side corner, although the field enhancement is larger for the gold nano-aperture. Hence, the fluorescence intensity is weak at the aperture side, and the highly efficient fluorescent enhancement occurs mostly at the central region for the pure gold nano-aperture.

In contrast to the Au nano-aperture, the antenna quantum yield of the Au/Si hybrid antenna remains high in a large region at the same *x*-position—even for the emitters that are located close to the side corners. We note that for the pure silicon aperture, the antenna quantum yield is higher due to low loss, but the field enhancement factor is low (Figure S5). These conclusions are similar to previous reports [24,31]. In addition, the relative photoluminescence (PL) enhancement is estimated approximately based on the product of excitation field enhancement and the antenna quantum yield. The calculated PL enhancements at the positions that are mentioned above are plotted in Figure 2g,h. These results imply that the Au/Si hybrid antenna has better surface-enhanced fluorescence performance than the Au nano-aperture antenna.

The antenna quantum yields for both the gold and hybrid aperture antennas show similar vertical variation of the emitter position, i.e., they decrease with increasing positions along the *z*-direction. In particular, signal collection only from the bottom side shows that the relative quantum yield is more sensitive to the dipole position in the *z*-direction (Figures S3 and S4). Hence, the high efficiency of the fluorescent emission occurs mainly within the shallow layer that is close to the water-glass interface when both the excitation and collection are from the bottom side. Finally, by considering the field enhancement and quantum yield together as above, an efficient PL enhancement occurs in a shallow region that is close to the water-glass interface, which is helpful for reducing the observation volume to beyond the diffraction limit.

Having preliminarily demonstrated the advantages of the hybrid nano-aperture antenna, we next asked how the aperture size or layer thickness affects the enhancement effects. We calculated the far-field radiative rate enhancement and the antenna quantum yields as a function of the aperture diameter size. All of the calculations were executed for an emitter that was located at the center of a nano-aperture 10 nm above the silica surface, as above. The dipole orientates along the *x*-direction. We noted that the fluorescence enhancement is dependent on the dipole orientation [32]. For example, the antenna quantum yield of the z-orientated dipole is lower than the in-plane dipoles, and the *z*-component of electric filed is very weak under illumination of in-plane polarized light (the representative calculations are shown in supplemental materials). These factors result in much less signal from the *z*-orientated dipole. Hence, we focus our attention on the *x*-orientated dipole due to the aperture symmetry.

**Figure 3:**
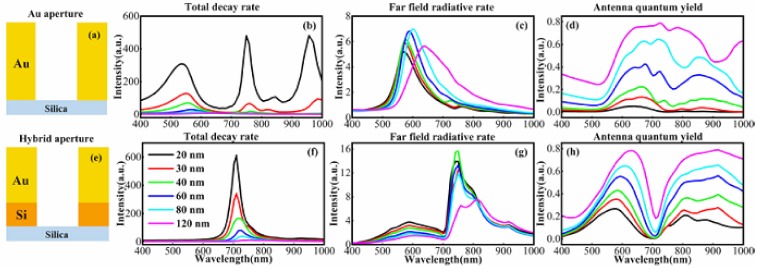
The enhancement effects are dependent on the aperture size of the antenna: (**b**) total decay rate enhancements; (**c**) far-field radiative rate enhancements; and (**d**) antenna quantum yields of (**a**) the Au nano-aperture antenna with diameters from 20 to 120 nm. Panels (**f**–**h**) show the same corresponding plots for (**e**) the Au/Si hybrid nano-aperture antenna with diameters from 20 to 120 nm, respectively. The thickness of the Au layer is 100 nm for gold aperture, and the hybrid structure consists of an 80 nm Au layer and a 20 nm Si layer.

Figure 3 shows that the hybrid aperture antenna has different enhancement features versus pure gold nano-aperture as the aperture size varies from 20 to 120 nm. For the Au nano-aperture that is shown in Figure 3c, the far-field radiative rate enhancement factor generally increases with the increasing aperture diameter. It then decreases when the diameter reaches or is over 80 nm. Moreover, the maximum radiative rate always redshifts, and the half width at half maximum become broader. Concurrently, the antenna quantum yield increases monotonously with increasing diameter, due to increasing separation between the emitter and metal surface.

Although smaller gold apertures have higher localized field enhancement, the antenna quantum yield is lower. Hence, the high enhancement effect occurs for the gold apertures with diameters of about 120 nm. (Larger apertures have low total fluorescent enhancement due to much lower field enhancement; data are not shown here). This simulation result agrees nicely with previous experimental studies [33].

The Au/Si hybrid apertures (Figure 3g) have enhanced far-field radiative rates with the increasing aperture diameter; this is independent of wavelength. Moreover, the radiative enhancement is higher than the gold apertures, which is consistent with the field enhancement calculations that are shown in Figure 2. The quantum yield also increases with the increasing aperture diameter (Figure 3h). The hybrid aperture still has a considerable antenna quantum yield, even down to 20 nm. This implies that the hybrid aperture has better performance beyond the diffraction limit for single molecule analysis, even at higher concentrations. The hybrid nano-aperture can also detect molecular fluorescence at the near-infrared region with a small size aperture. The dip in quantum yield at 700 nm is attributed to the surface mode between the metal and dielectric layers. Further calculations demonstrate that the hybrid nano-apertures with Si layer thickness from 20 to 60 nm have good surface enhancement effect (data is not shown here) that allows for a wide tolerance to experimentally fabricate such hybrid antennae.

In surface-enhanced spectroscopy, an important strategy to optimizing the enhancement effect is to construct plasmonic gap configurations [34]. The plasmon gap mode was first introduced into nano-aperture antenna by Lu et al. via a bowtie structure for molecule fluorescence analysis [35]. Later, the nano-aperture with plasmonic gap for molecule fluorescence analysis was further improved greatly via an antenna-in-box device by Punj et al. [36] Following this strategy, we introduce the dielectric nanogap structures into the Au/Si hybrid nano-aperture to further optimize the enhancement effect.

**Figure 4:**
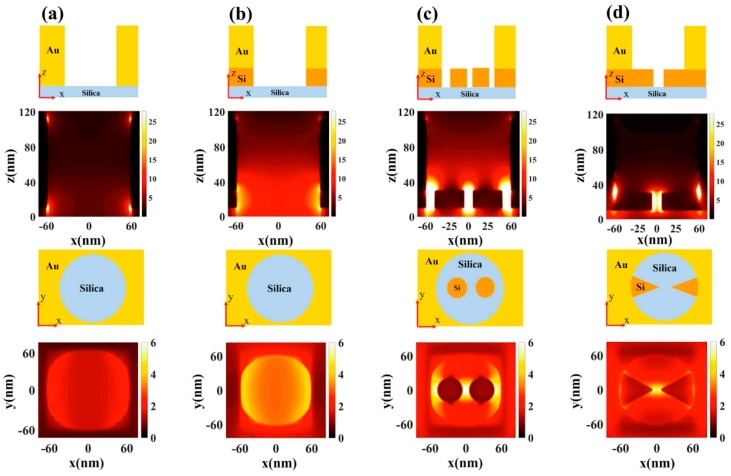
Electric field distribution |E/E_0_|^2^ and corresponding schematics in the *xz*-plane and the *xy*-plane for (**a**) Au nano-aperture at 573 nm; (**b**) Au/Si hybrid nano-aperture at 785 nm; (**c**) Au/Si hybrid nano-aperture with a silicon disk dimer at 758 nm; and (**d**) Au/Si hybrid nano-aperture with a silicon triangle gap at 758 nm. The diameters of all of the apertures are 120 nm and the silicon thickness is 20 nm. The disks’ diameter in (**c**) is 40 nm, and the silicon gaps are 10 nm both in (**c**,**d**).

Figure 4 shows four kinds of nano-apertures simulated and compared to demonstrate the nanogap features. Two Si nanogaps are proposed to collaborate with a 120-nm diameter aperture. The first is a nanogap composed of two silicon disks. The thickness of the silicon disk is 20 nm, and the gap distance is 10 nm (Figure 4c). The second is a nanogap consisting of two silicon triangles—the thickness is 20 nm and the gap distance is 10 nm (Figure 4d). We calculate the electromagnetic field distribution in both the *xz*-plane and the *xy*-plane (Figure 4). Our simulations show that the field enhancement in the disk dimers nanogap is ~4-fold higher in magnitude than that of the pure gold aperture. The field enhancement could be optimized by adjusting the nanostructure parameters, e.g., smaller gap separation or sharper apex. The high field enhancement is very localized around the Si nanogap. This is useful for single molecule analysis at high concentrations.

**Figure 5:**
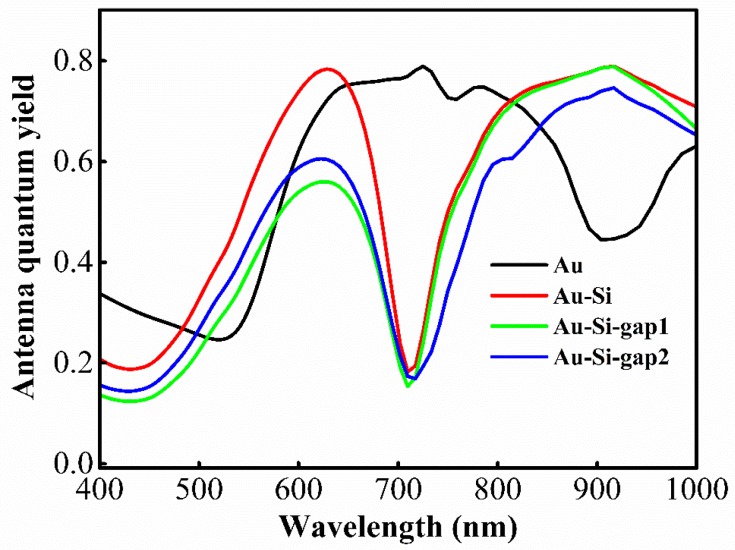
Antenna quantum yield as a function of wavelength from 400 nm to 1000 nm for four nano-aperture antennas shown in Figure 4. All of the calculations are performed for a dipole at the position (0, 0, and 10 nm). The Au-Si-gap1 and Au-Si-gap2 refer to the structures in Figure 4c,d, respectively.

We also calculated the antenna quantum yields as a function of wavelength (Figure 5); here, an *x*-orientated dipole is placed at the center of the structures for all of the calculations. The antenna quantum yield of the nano-apertures with Si nanogaps is comparable to that of bare nano-apertures. Therefore, the hybrid apertures with a Si nanogap can greatly enhance the molecular fluorescence. There is always a dip near 700 nm (Figure 5) due to the surface mode between the metal and the dielectric layers. This intrinsic spectral feature could offer dual color analysis [7].

Another important strategy to optimize the surface enhancement effect is to control the emission direction of the molecule’s fluorescence, as first shown experimentally by Aouani et al. for a gold nano-aperture antenna [37]. The periodic grating structures usually surround the nano-aperture to both enhance the excitation rate and converge the emission for high collection efficiency [38,39]. Meanwhile, the silicon layer can be replaced with a lower loss dielectric material in the spectral region (e.g., GaP [40] in visible region). This would increase the antenna quantum yield. Experimentally, this hybrid nano-aperture antenna with or without Si nanogap is achievable via nanofabrication; experimental works are underway.

## 4. Conclusions

In summary, hybrid metal-dielectric nano-aperture antennas were investigated and compared theoretically by employing the FDTD method. The hybrid nano-apertures show better surface enhancement effect, rather than pure metal nano-apertures. The hybrid nano-apertures show a uniform field distribution within the apertures and higher antenna quantum yield than pure gold nano-apertures. The spectral feature of hybrid nano-apertures is independent from the aperture size and it shows a high enhancement effect in the near-infrared region even with small size aperture. Moreover, the hybrid nano-apertures show two high enhancement bands. This intrinsic feature benefits dual color analysis. Furthermore, the hybrid nano-apertures with dielectric gaps are useful for larger enhancement effects. The hybrid nano-aperture is fully adaptable to large-scale availability, and a Si layer thickness of 20 to 60 nm offers a good enhancement effect that facilitates a wide fabrication tolerance. The hybrid antennae will significantly improve the efficiency of surface-enhanced fluorescence for sensitive biosensing and molecular fluorescence applications.

## Supplementary Materials

The following are available online at http://www.mdpi.com/1996-1944/11/8/1435/s1, Figure S1: Representative antenna quantum yields of the horizontal dipole along the *z*-, *y*-direction and the vertical dipole along the *z*-direction within (a) the Au aperture and (b) the Au/Si hybrid aperture at the position (0, 0, 10 nm; the origin is at the center of water-glass interface), correspondingly, Figure S2: Field distribution (a)|Ex|2, (b) |Ey|2 and (c) |Ez|2 in the *xz*-plane and *xy*-plane for Au nano-aperture antenna at a wavelength of 573 nm, Au/Si hybrid nano-aperture antenna at a wavelength of 785 nm illuminated by a plane wave with *x*-polarization from the bottom, Figure S3: Radiative rate of bottom collection (Prad-glass) and full integrated (Prad) for (a) the Au nano-aperture and (b) the Au/Si hybrid nano-aperture, Figure S4: Position-dependent quantum yields (the sizes of red disks represent the relative value of quantum yield at the positions correspondingly) (a,c) at a wavelength of 660 nm for Au nano-aperture antenna, (b,d) at a wavelength of 814 nm for Au/Si hybrid nano-aperture antenna; (e,f) show quantum yields with different *x*- and *z*-positions in the way of (a–b) full integrated and (c,d) bottom collection, correspondingly, Figure S5: Field distribution |E/E_0_|2 in the *xz*-plane and *xy*-plane for (a) Au nano-aperture antenna at a wavelength of 573 nm, (b) Au/Si hybrid nano-aperture antenna at a wavelength of 785 nm, (c) Si nano-aperture antenna at a wavelength of 725 nm illuminated by a plane wave with *x*-polarization from the bottom. (d) shows the antenna quantum yields of structures above which all are calculated for a dipole located at position (0, 0, 10 nm), Figure S6: Aperture size of the antenna-dependent enhancement effects. (b) Total decay rate enhancements, (c) Far-field radiative rate enhancements, (d) Non-radiative rate, (e) Antenna quantum yields of (a) the Au nano-aperture antenna with diameters from 20 to 120 nm. (g,h) show the same corresponding plots for (f) the Au/Si hybrid nano-aperture antenna with diameters from 20 to 120 nm, respectively. The thickness of the Au layer is 100 nm for gold aperture, and the hybrid structure consists of an 80 nm Au layer and a 20 nm Si layer.
