# Supplementary material for: Hybrid Metal-Dielectric Nano-Aperture Antenna for Surface Enhanced Fluorescence
Source: Materials (Basel). 2018 Aug 14;11(8):1435. doi: 10.3390/ma11081435 (PMC6119926; doi:10.3390/ma11081435)
Supplement: Supplementary file 1 [file materials-11-01435-s001.pdf]

materials

Supplement

Hybrid metal-dielectric nano-aperture antenna for surface enhanced fluorescence

Guowei Lu,[1,2,*] Jianning Xu[1], Te Wen[1], Weidong Zhang[1], Jingyi Zhao[1], Aiqin Hu[1], Grégory Barbillon[3] and Qihuang Gong[1,2]

1 State Key Laboratory for Mesoscopic Physics & Collaborative Innovation Center of Quantum Matter, School of Physics, Peking University, Beijing 100871, China; jnxu@pku.edu.cn (J.X.); wente@pku.edu.cn (T.W.); weidongzhang@pku.edu.cn (W.Z.); jingyi.zhao@pku.edu.cn (J.Z.); aiqinhu@pku.edu.cn (A.H.); qhgong@pku.edu.cn (Q.C.)
2 Collaborative Innovation Center of Extreme Optics, Shanxi University, Taiyuan 030006, China
3 EPF—École d'INGénieurs, 3 bis rue Lakanal, 92330 Sceaux, France; gregory.barbillon@epf.fr
* Correspondence: guowei.lu@pku.edu.cn

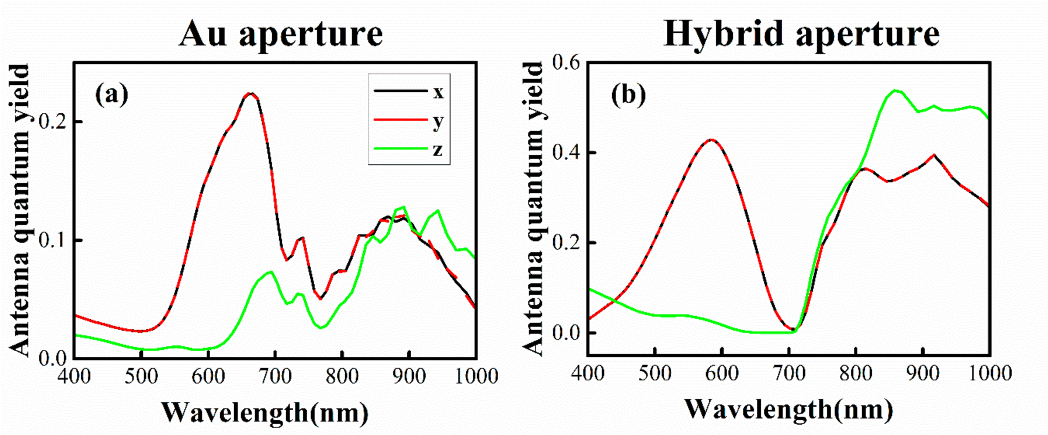

Figure S1. Representative antenna quantum yields of the horizontal dipole along the *z*-, *y*-direction and the vertical dipole along the *z*-direction within (**a**) the Au aperture and (**b**) the Au/Si hybrid aperture at the position (0, 0, 10 nm; the origin is at the center of water-glass interface), correspondingly.

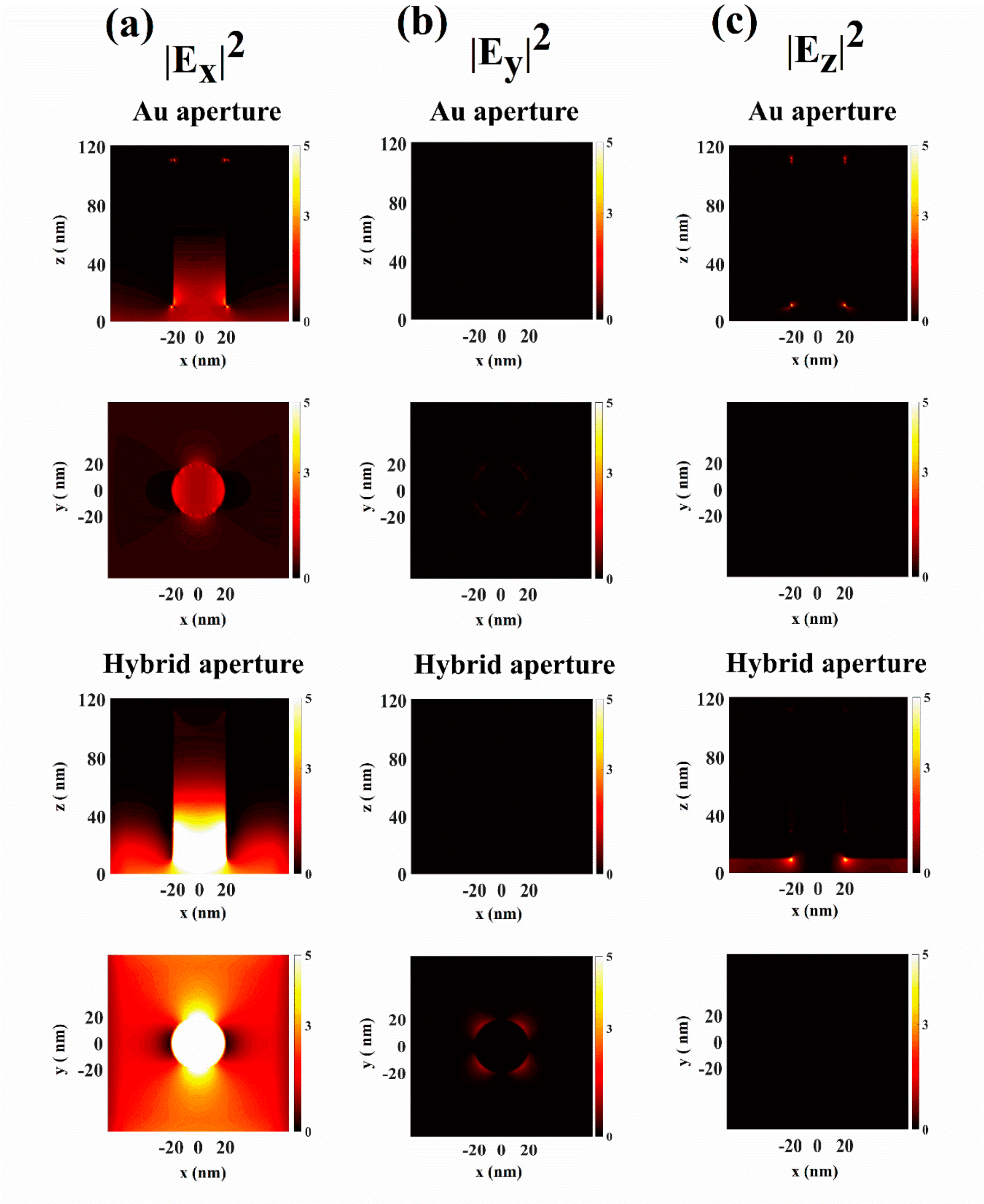

Figure S2. Field distribution (**a**) $|E_x|^2$, (**b**) $|E_y|^2$ and (**c**) $|E_z|^2$ in the *xz*-plane and *xy*-plane for Au nano-aperture antenna at a wavelength of 573 nm, Au/Si hybrid nano-aperture antenna at a wavelength of 785 nm illuminated by a plane wave with *x*-polarization from the bottom.

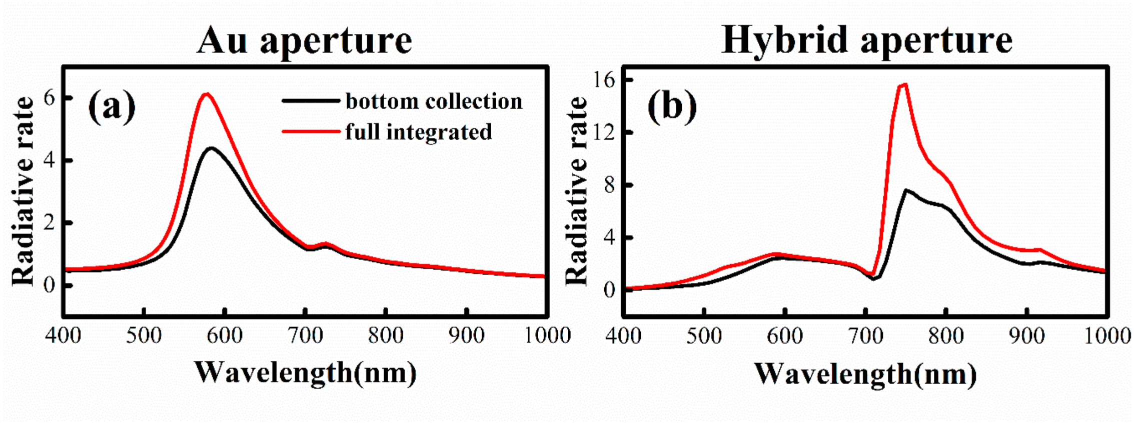

Figure S3. Radiative rate of bottom collection ($P_{rad\text{-}glass}$) and full integrated (P_{rad}) for (**a**) the Au nano-aperture and (**b**) the Au/Si hybrid nano-aperture.

The "bottom connection" ($P_{rad\text{-}glass}$) means that the radiated emission is collected from the bottom, i.e. the glass side. The "full integrated" (P_{rad}) means to collect all far-field radiated emission, it can be obtained by integrating the Poynting vector over closed surfaces that contain the nanoantenna and dipolar source.

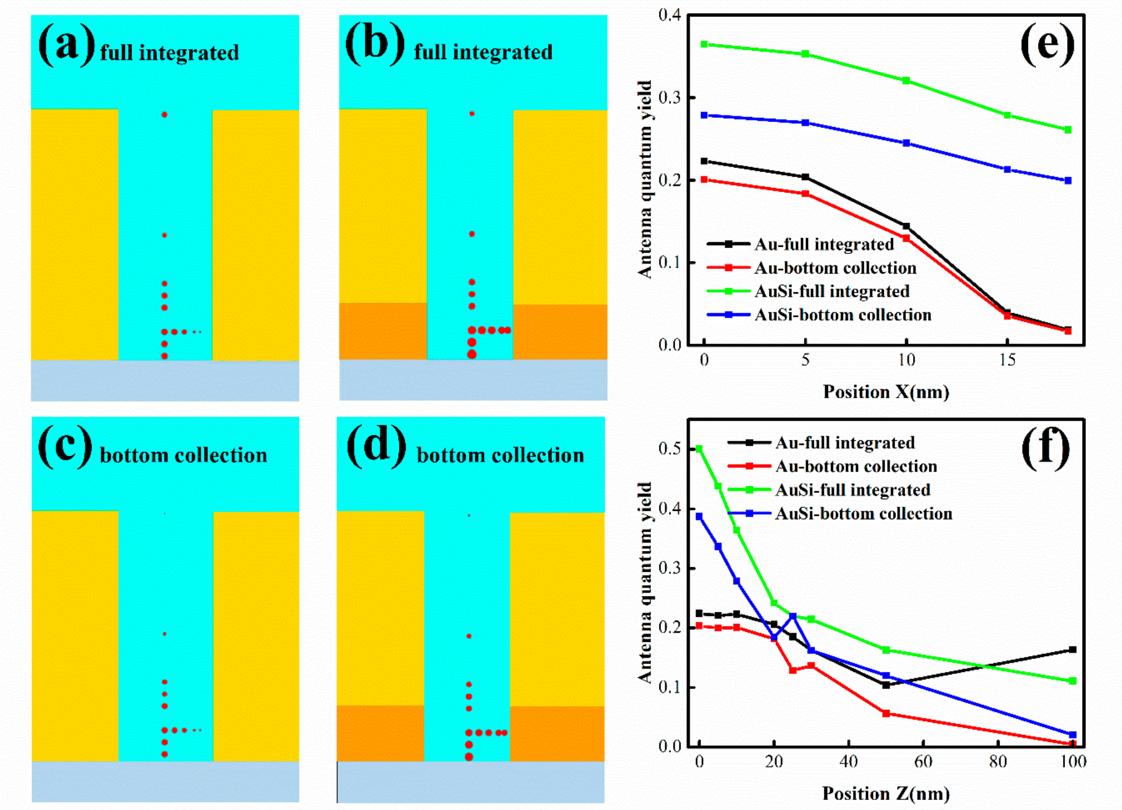

Figure S4. Position-dependent quantum yields (the sizes of red disks represent the relative value of quantum yield at the positions correspondingly) (**a**), (**c**) at a wavelength of 660 nm for Au nano-aperture antenna, (**b**), (**d**) at a wavelength of 814 nm for Au/Si hybrid nano-aperture antenna; (**e**) and (**f**) show quantum yields with different *x*- and *z*-positions in the way of (**a**)–(**b**) full integrated and (**c**)–(**d**) bottom collection, correspondingly.

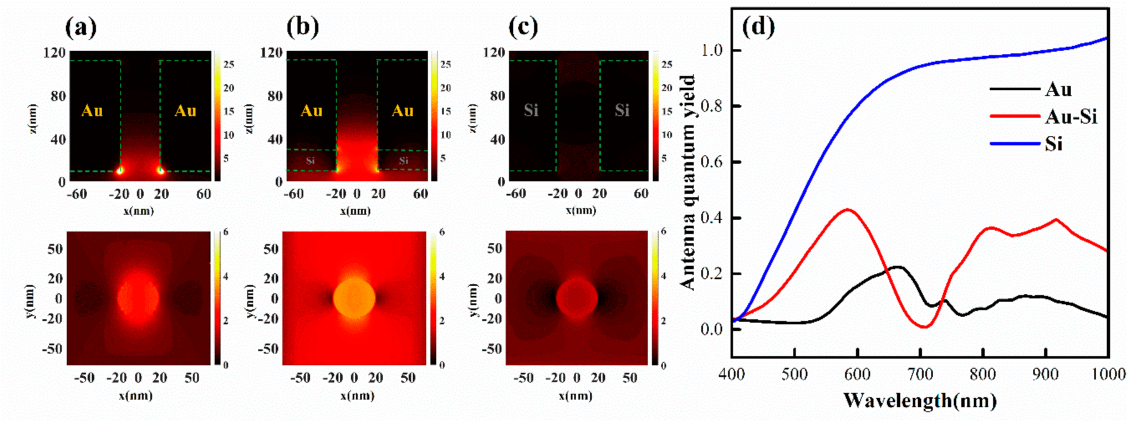

Figure S5. Field distribution $|E/E_0|^2$ in the *xz*-plane and *xy*-plane for (**a**) Au nano-aperture antenna at a wavelength of 573 nm, (**b**) Au/Si hybrid nano-aperture antenna at a wavelength of 785 nm, (**c**) Si nano-aperture antenna at a wavelength of 725 nm illuminated by a plane wave with *x*-polarization from the bottom. (**d**) shows the antenna quantum yields of structures above which all are calculated for a dipole located at position (0, 0, 10 nm).

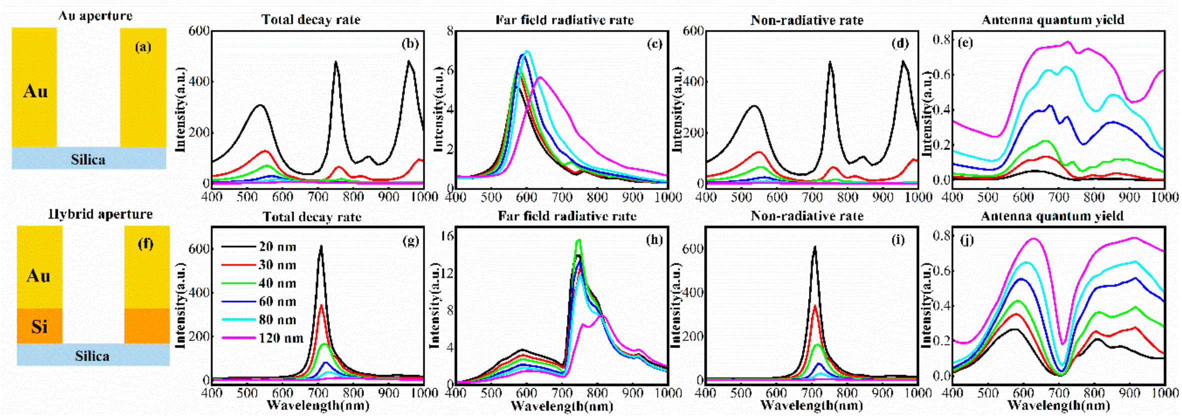

Figure S6. Aperture size of the antenna-dependent enhancement effects. (**b**) Total decay rate enhancements, (**c**) Far-field radiative rate enhancements, (**d**) Non-radiative rate, (**e**) Antenna quantum yields of (**a**) the Au nano-aperture antenna with diameters from 20 to 120 nm. (**g**)–(**h**) show the same corresponding plots for (**f**) the Au/Si hybrid nano-aperture antenna with diameters from 20 to 120 nm, respectively. The thickness of the Au layer is 100 nm for gold aperture, and the hybrid structure consists of an 80 nm Au layer and a 20 nm Si layer.

© 2018 by the authors. Submitted for possible open access publication under the terms and conditions of the Creative Commons Attribution (CC BY) license (http://creativecommons.org/licenses/by/4.0/).
